# Supplementary material for: Early NK-cell and T-cell dysfunction marks progression to severe dengue in patients with obesity and healthy weight
Source: Nat Commun. 2025 Jul 1;16:5569. doi: 10.1038/s41467-025-60941-9 (PMC12214611; doi:10.1038/s41467-025-60941-9)
Supplement: Supplementary file 1 — Supplementary Information [file 41467_2025_60941_MOESM1_ESM.pdf]

Supplementary Fig.S1

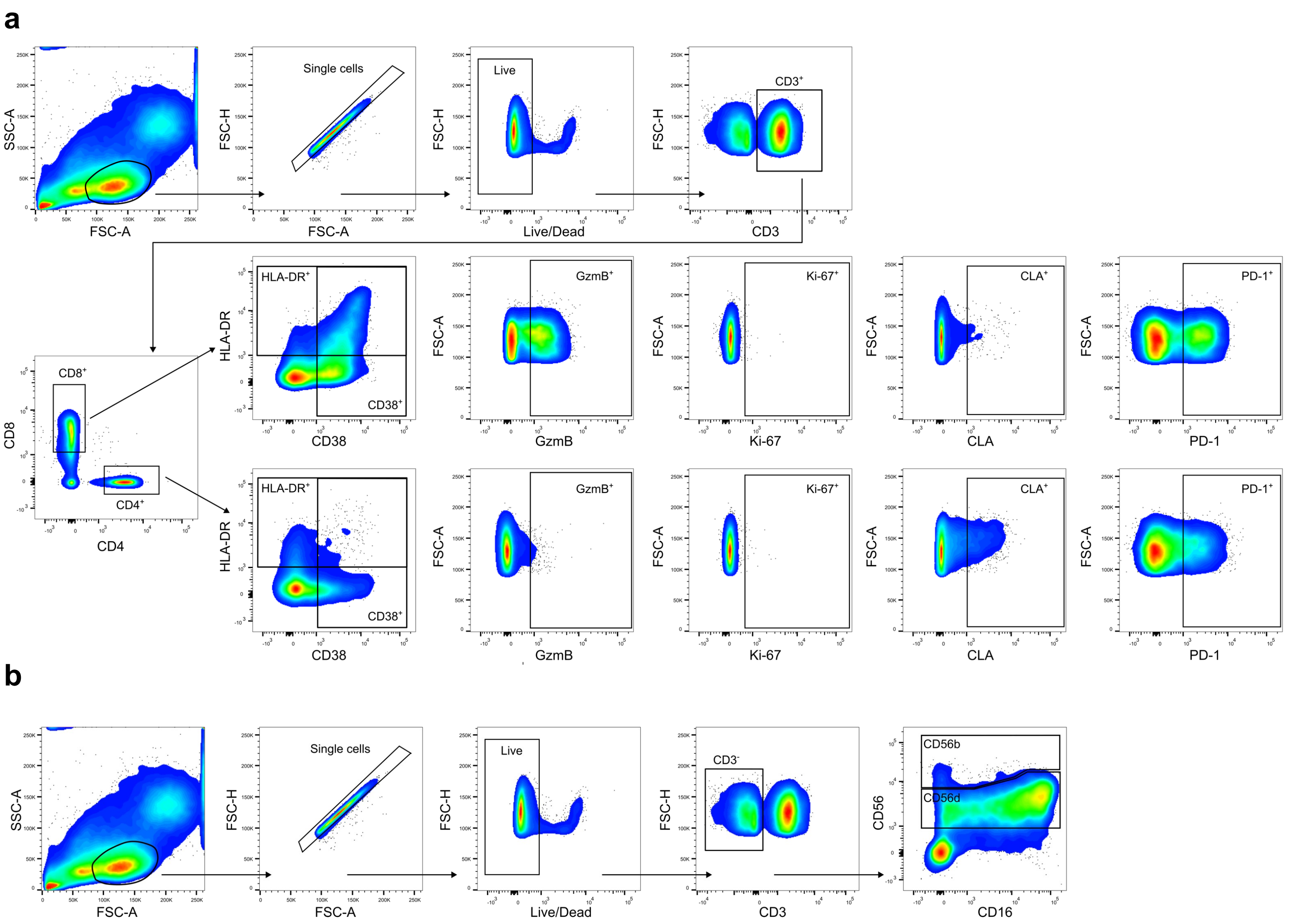

Supplementary Fig.S1. Gating strategy to identify CD4⁺ T-cells, CD8⁺ T-cells and NK-cell subsets. (a-b) Representative flow cytometry staining showing the gating strategy used to identify CD4⁺ and CD8⁺ T-cells and expression of the indicated markers (a); total and CD56^{dim} (CD56d) and CD56^{bright} (CD56b) NK-cells (b).

Supplementary Fig.S2

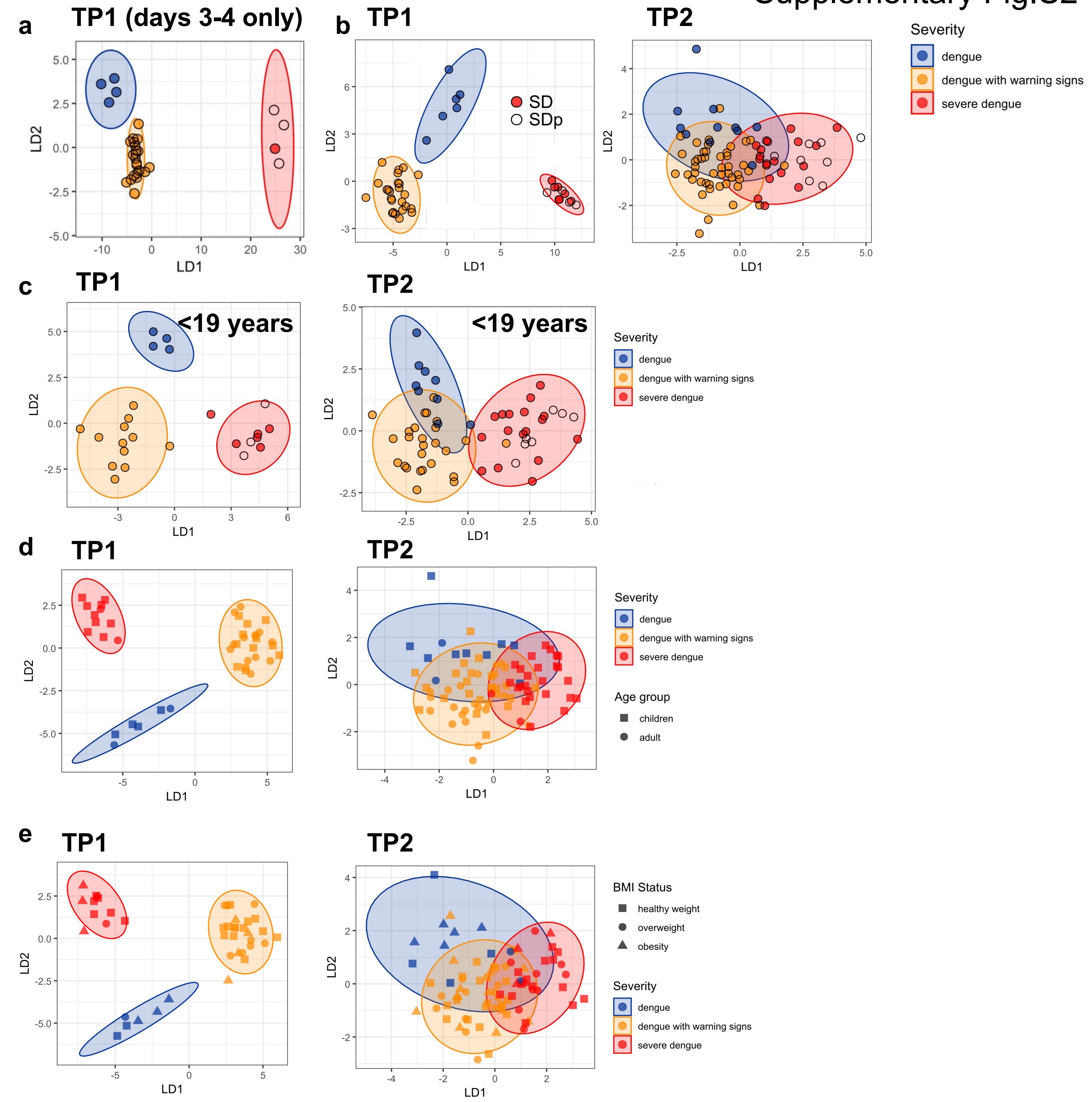

Supplementary Fig.S2. Distinct T and NK-cell profiles associate with dengue severity. Linear discriminant analysis (LDA) of T and NK-cell flow cytometry data from PBMCs of patients with dengue (D), dengue with warning signs (DWS) and severe dengue (SD) are shown in blue, yellow and red, respectively. (a) Data is shown only for patients at days 3-4 (TP1; N=30). In b-e data is shown for all patients stratified based on the indicated criteria, and at the two timepoints (TP) of disease [TP1 (N=42) and TP2 (N=84)]. (b) Data for patients from the SD group who progressed to SD during the study are shown using empty circles (SD progressors, SDp: N=4 which includes N=2 at day 4, N=1 at day 3 and N=1 at day 2), while data for patients who had already progressed to SD at admission (SD: N=7 which includes N=1 at day 4 and N=6 at day 5) are shown with filled red circles. (c) Data is shown only for paediatric patients <19 years, with SDp and SD highlighted as explained above (N=97). (d) Data is shown for all patients stratified by age. Data for paediatric (< 19 years) and adult patients (≥ 19 years) are shown in squares and circles, respectively. (e) Data is shown for all patients stratified by BMI. Patients with healthy weight (HW), overweight (OW) and obesity (OB) are shown in squares, circles and triangles, respectively. Ellipses represent 95% confidence intervals. LD1 and LD2 were derived using all features shown in Fig.1d-i.

Supplementary Fig.S3

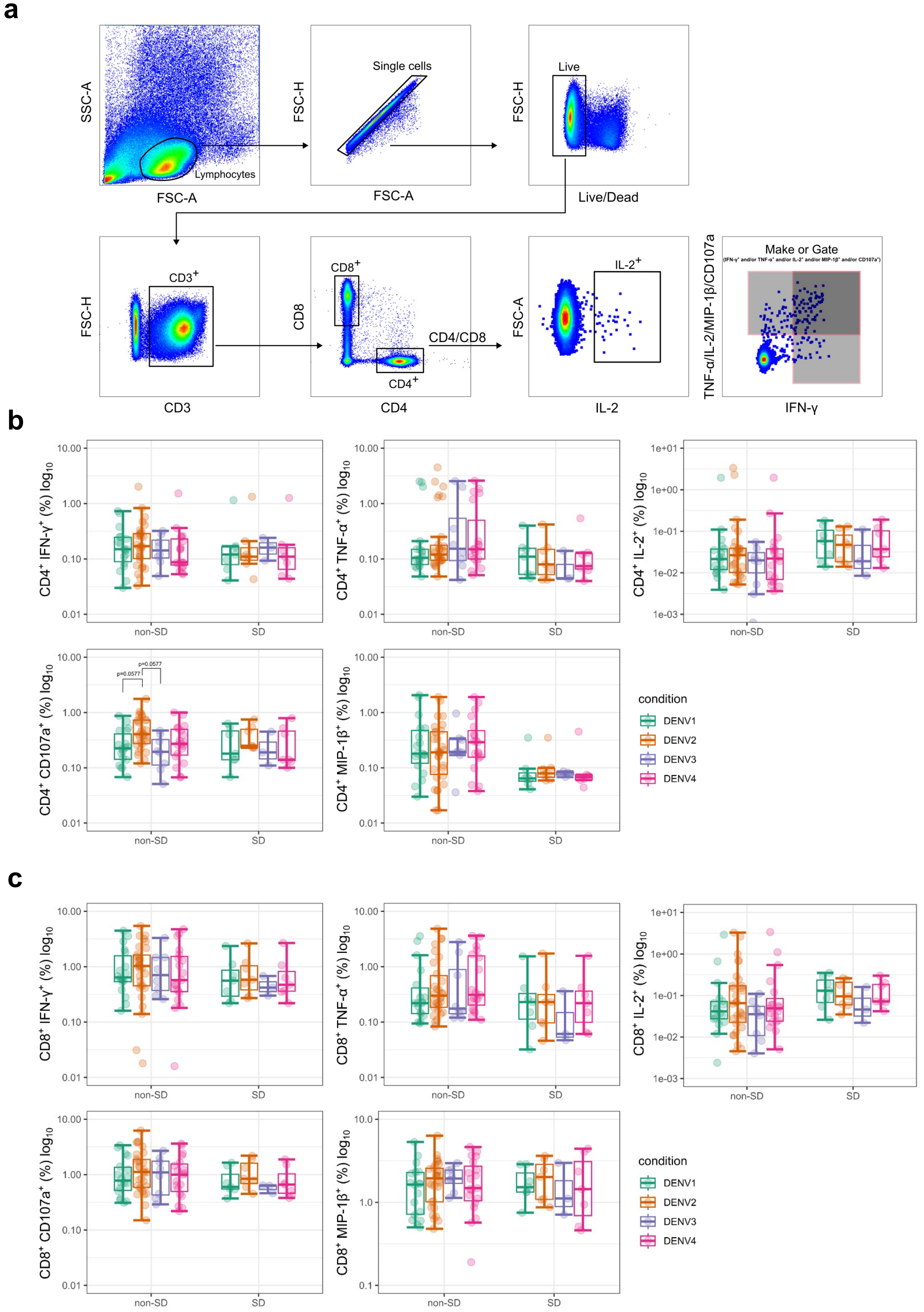

Supplementary Fig.S3. DENV NS3-specific T-cell response. (a) A representative staining depicting the boolean gating strategy used to identify cytokine$^+$ and/or CD107a$^+$ T-cells is shown for CD8$^+$ T-cells; the same approach was used for CD4$^+$ T-cells. (b-c) Individual cytokine responses by CD4$^+$ (b) and (c) CD8$^+$ T-cells following NS3 DENV1-4 peptide stimulation is shown for non-severe dengue (non-SD) and severe dengue (SD) patients at TP2 (N=37). The middle line in each box represents the median with IQR. Error bars represent max/min value +/- 1.5*IQR. Statistics calculated using one-way ANOVA with Benjamini-Hochberg correction.

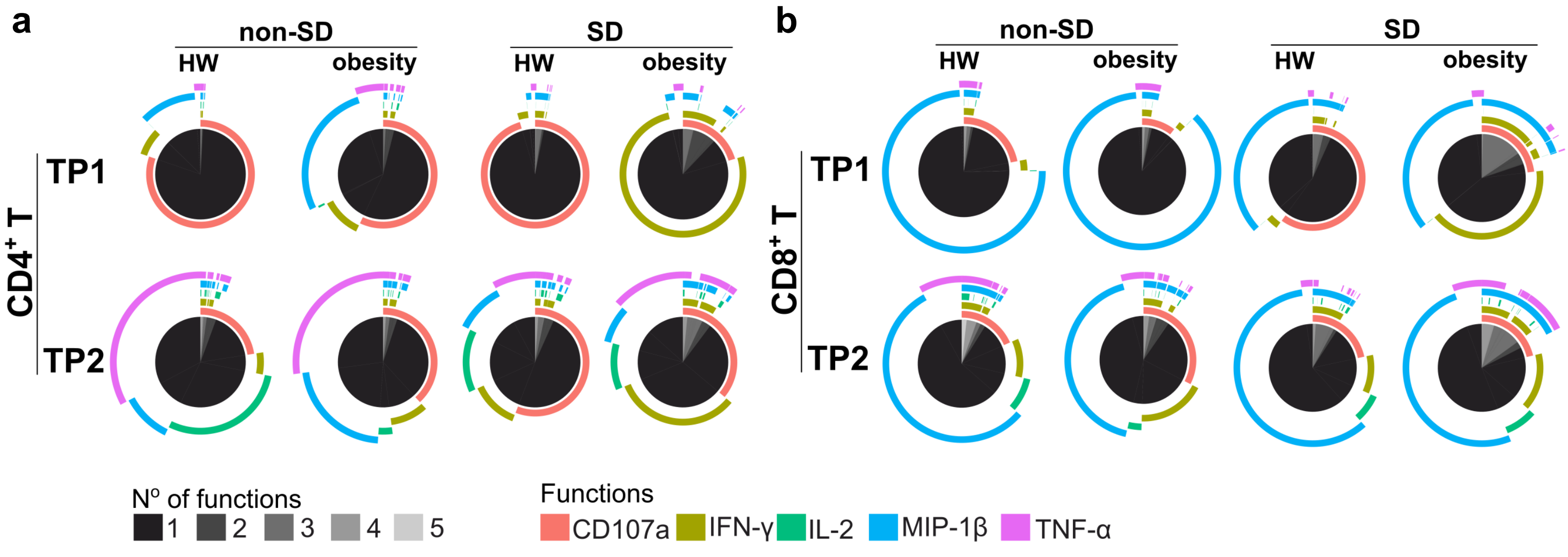

Supplementary Fig.S4. Assessment of DENV2-specific T-cell function during DENV2 infection. (a, b) Pie charts showing the number of functions simultaneously exhibited by $CD4^+$ (a) and $CD8^+$ T-cells (b) following NS3 DENV2 peptide stimulation at TP1 (N=24) and TP2 (N=37). The different shades of grey represent the range of 1-5 functions, the outer arcs indicate the specific functions (IFN-γ/TNF-α/IL-2/MIP-1β/CD107a) as defined by Boolean gating.

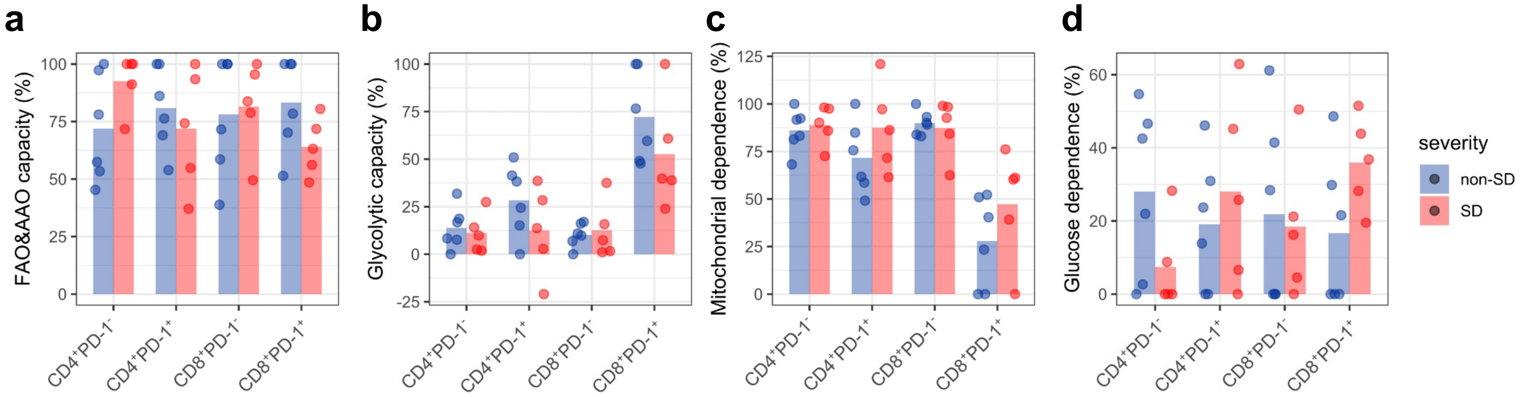

Supplementary Fig.S5. Metabolic activity measured by SCENITH. (a) Fatty acid and amino acid oxidation (FAO&AAO) capacity, (b) glycolytic capacity, (c) mitochondrial dependence, and (d) glucose dependence (N=11). Bar plots showing the mean values for specific cell subsets, with colours indicating non-severe dengue (non-SD; blue) or severe dengue (SD; red) patient groups. SCENITH was performed and capacities and dependencies were calculated according to Arguello et al.[32] and Luscombe et al.[33].

Supplementary Fig.S6

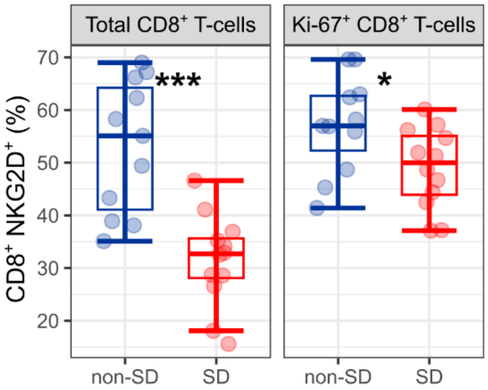

Supplementary Fig.S6. Decreased NKG2D expression in CD8⁺ T-cells of SD patients. Frequency of total or Ki-67⁺ CD8⁺ T-cells expressing NKG2D in non-severe dengue (non-SD; N=11) and severe dengue (SD; N=12) patients. Statistics were calculated by Wilcoxon test. The middle line in each box represents the median with IQR. Error bars represent max/min value +/- 1.5*IQR.

Supplementary Fig.S7

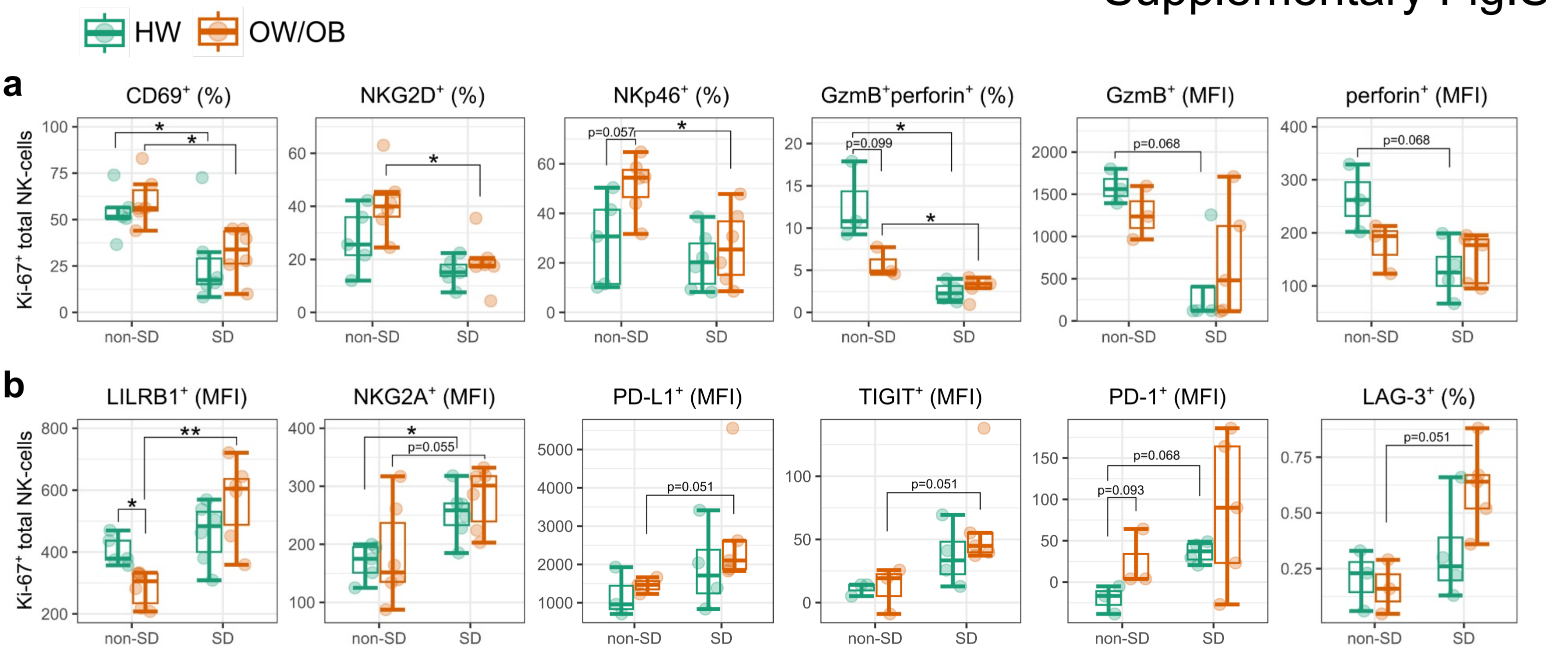

Supplementary Fig.S7. Phenotype of Ki-67⁺ total NK cells in SD and non-SD patients stratified by BMI group. (a, b) Expression of activating (a; N-23) and inhibitory receptors (b; N=15) by Ki-67⁺ total NK-cells in HW (green) and OW/OB (orange) non-SD and SD patients. Statistics were calculated by one-way ANOVA with Benjamini-Hochberg correction. The middle line in each box represents the median with IQR. Error bars represent max/min value +/- 1.5*IQR.

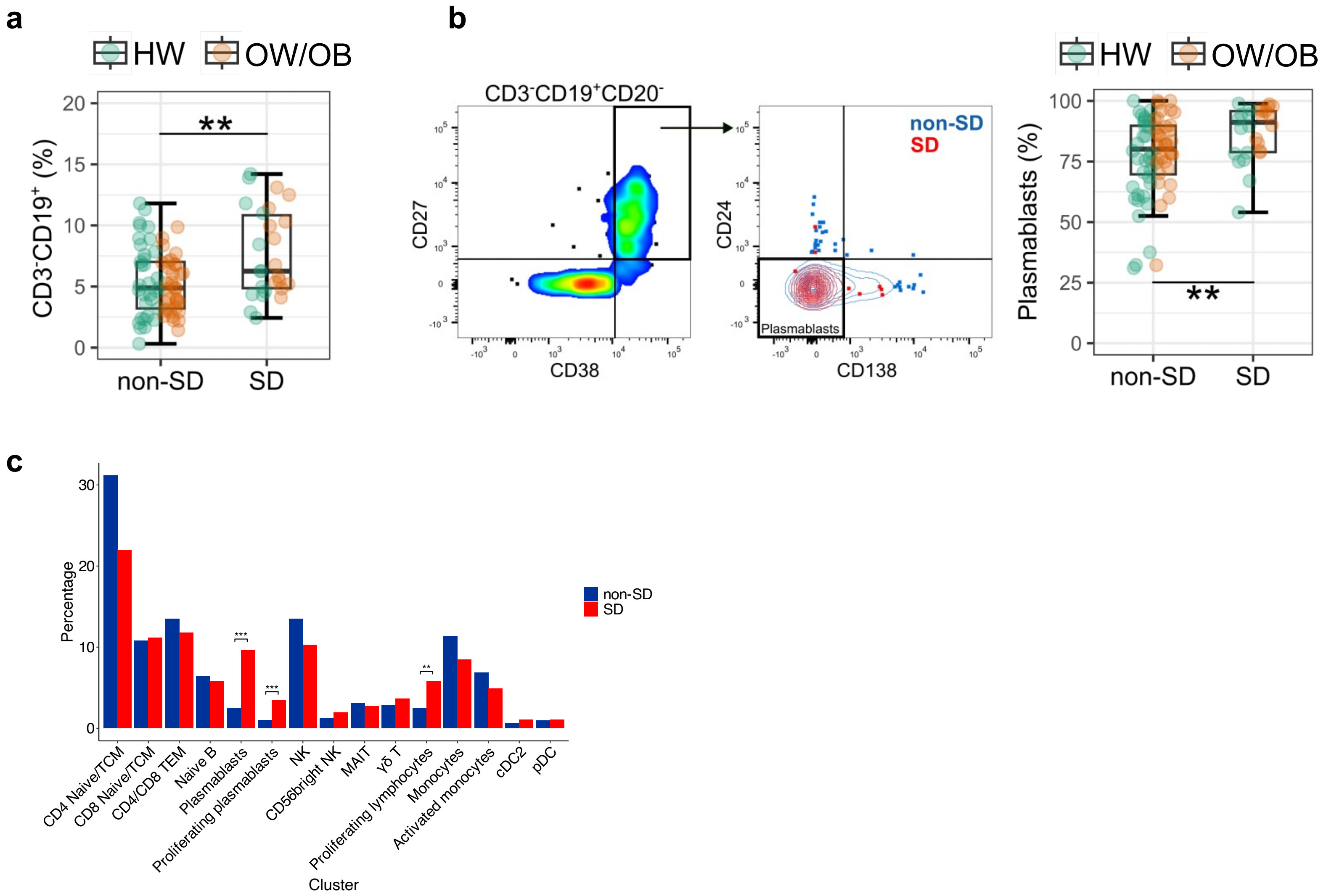

Supplementary Fig.S8. Increased frequency of B-cells and plasmablasts in SD. (a) Frequency of $CD3^{-}CD19^{+}$ cells as assessed by flow cytometry. (b) Flow cytometry gating strategy and frequency of plasmablasts in non-severe dengue (non-SD) and severe dengue (SD) patients [HW (green) and OW/OB (orange)]. Data from N=94 dengue patients at TP2 (N=68 non-SD; N=26 SD). Statistics were calculated by Wilcoxon test. The middle line in each box represents the median with IQR. Error bars represent max/min value +/- 1.5*IQR. (c) Percentage of cell types as assessed by scRNA-seq analyses (BD Rhapsody) of PBMC samples from N=24 patients (non-SD; N=12; SD; N=12). Differential abundance analysis was performed using edgeR, **p < 0.01, ***p < 0.001.

Supplementary Fig.S9

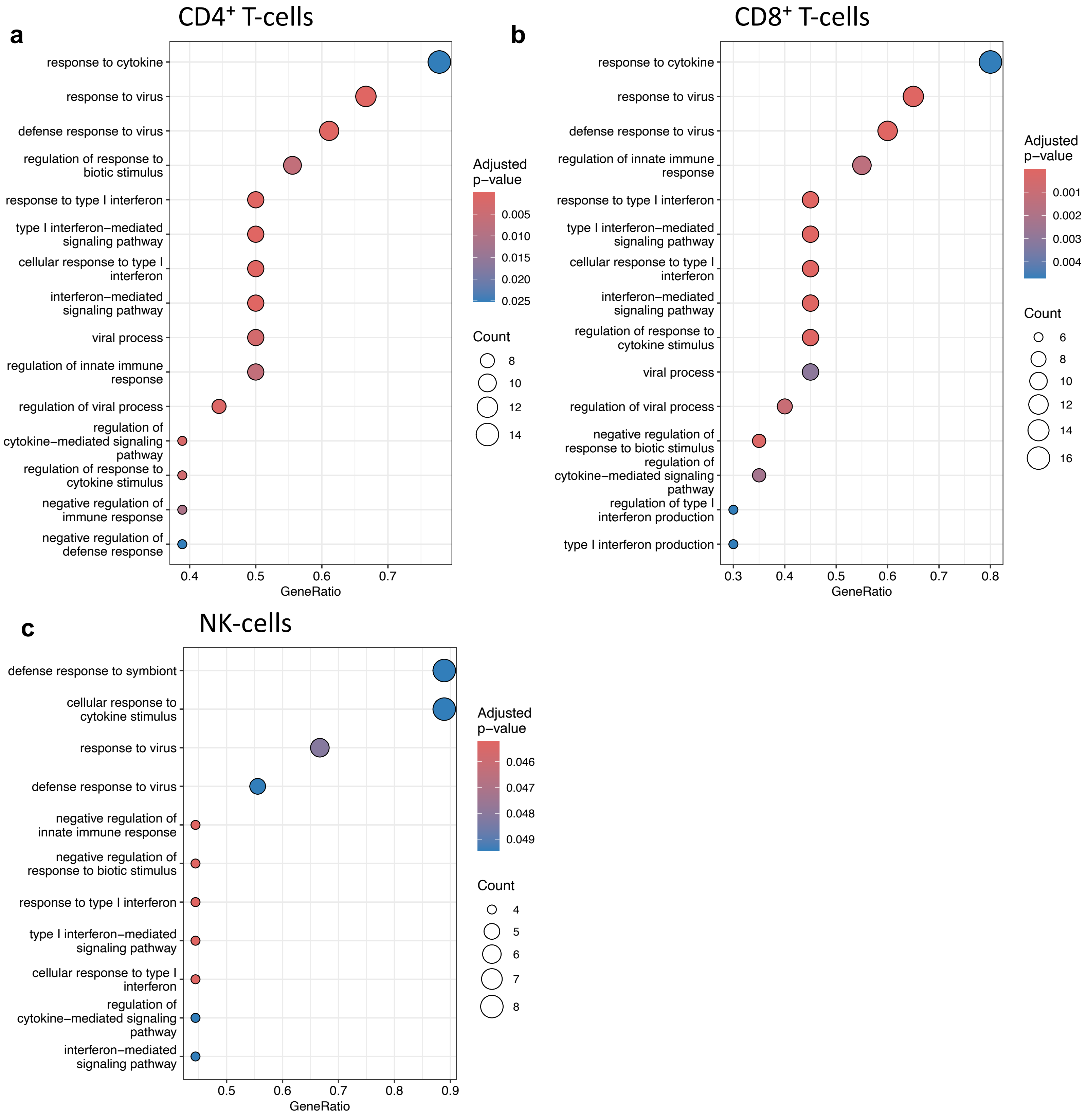

Supplementary Fig.S9. Impaired type-I IFN responses in SD. (a-d) Over-representation analysis of genes significantly downregulated in severe dengue (SD) vs non-severe dengue (non-SD) in: CD4+ T-cells (a), CD8+ T-cells (b) and NK-cells (c). All significant or top 15 non-redundant Gene Ontology terms and associated Benjamini-Hochberg adjusted p-values are shown. Count=number of differentially expressed genes (DEGs) in the gene set. GeneRatio=fraction of DEGs in the gene set. Data from N=24 dengue patients (N=12 non-SD; N=12 SD).

Supplementary Fig.S10

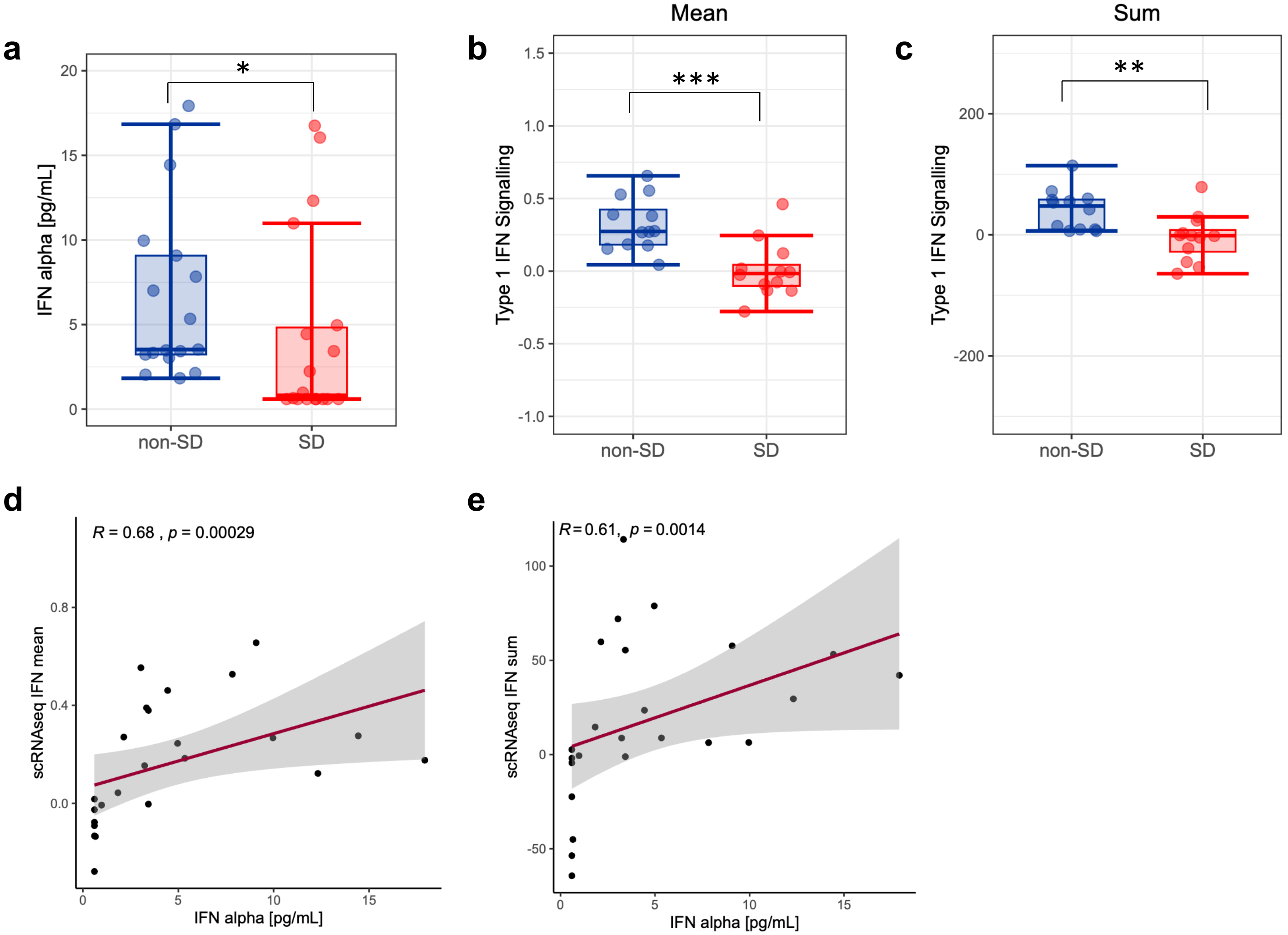

Supplementary Fig.S10. Decreased type-I IFN responses in SD. (a) Plasma levels of IFN-α (pg/ml) are shown for N=35 dengue patients (N=17 non-SD; N=18 SD) at TP1 (Thermo Fischer simplex IFN-α kit). (b, c) The extent of type-I IFN signalling in SD and non-SD patient PBMCs was determined by generating per cell type-I IFN signaling module scores from the scRNA-seq data Seurat "AddModuleScore" function using the genes in the "type I interferon-mediated signalling pathway" Gene Ontology term (GO:0060337). Per sample scores were generated by averaging (mean; b) or summing (c) individual cell scores. N=24 (Non-SD: N=12; SD: N=12). (d, e) Spearman correlation of IFN-α plasma levels and type-I IFN signalling scores determined by scRNA-seq and shown in b-c. Statistics for a-c were calculated by Wilcoxon test. The middle line in each box represents the median with IQR. Error bars represent max/min value +/- 1.5*IQR.

Supplementary Fig.S11

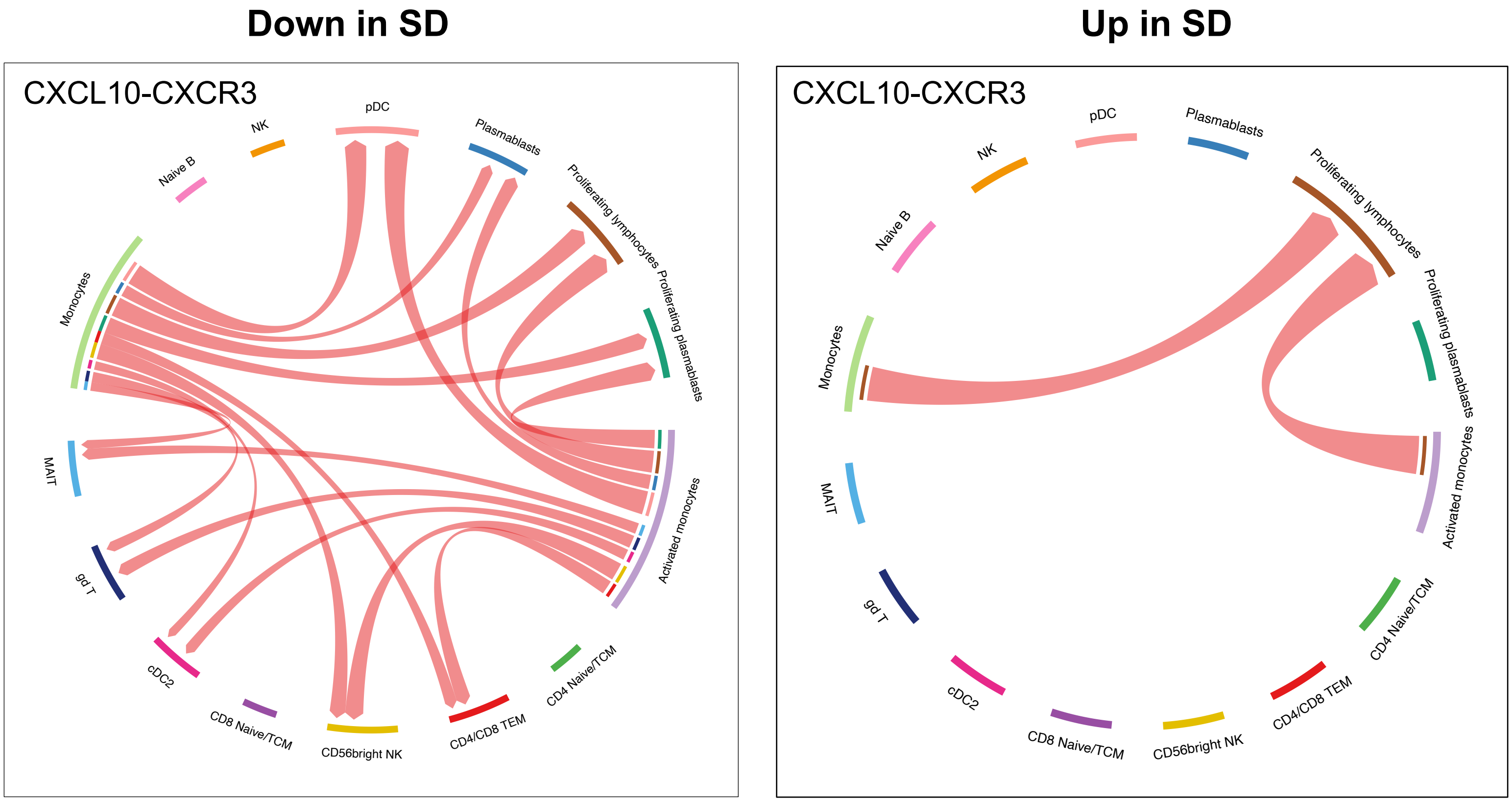

Supplementary Fig.S11. Decreased CXCL10-CXCR3 interactions in immune cells of SD patients. Cell-cell communication analysis was performed on scRNA-seq data using CellPhoneDB. Circos plots show CXCL10-CXCR3 interactions that were downregulated (left) or upregulated (right) in SD versus non-SD patients. N=24 (Non-SD: N=12; SD: N=12).

Supplementary Table S1. Details for the dengue patients enrolled in this study (N=124).

Number of patients (N)		Total (N=124)	Non-SD (N=94)	SD (N=30)
Age, N	Children (10-18 years)	82	56	26
	Adult (19-30 years)	42	38	4
	Median age, years (interval)	15 (10-30)	16.5 (10-30)	13 (10-30)
Sex, N	Female (F)	30	23	7
	Male (M)	94	71	23
	Ratio M:F	3.1	3.1	3.3
BMI groups, N	HW (median age: 15 years)	60	44	16
	OW/OB (median age: 15 years)	64	50	14
	Ratio HW:OW/OB	0.9	0.9	1.1
Serotype, N	DENV1	22	18	4
	DENV2	86	65	21
	DENV4	7	5	2
	N/D	9	6	3
	Ratio DENV2:DENV1/4	0.69	0.69	0.70
Infection, N	Primary	5	5	0
	Secondary	118	89	29
	N/D	1	0	1
Day of illness at enrolment, N (viraemia, log_{10}, copies/ml)	Day 1	1 (ND)	1 (ND)	0
	Day 2	24 (6.79)	22 (6.79)	2 (6.53)
	Day 3	64 (5.85)	58 (5.82)	6 (5.87)
	Day 4	17 (5.50)	13 (5.51)	5 (5.50)
	Day 5	16 (5.79)	0	16 (5.79)
	Median	3 (5.81)	3 (5.83)	5 (5.50)
Plasma leakage grade, N	Grade 0	60	59	1
	Grade 1	35	35	0
	Grade 2	29	0	29
WHO dengue classification, N	No warning signs	14	14	0
	With warning signs	80	80	0
	Severe	30	0	30

Abbreviations: SD, severe dengue; non-SD, non-severe dengue; BMI, body weight index; HW, healthy weight; OW/OB, overweight/obesity; WHO, World Health Organization; N/D, not determined/undetectable. Viraemia data are presented as median values.

Supplementary Table S2. List of custom genes from the BD Rhapsody gene panel.

Gene		
ACE2	*IKZF3*	*OAS1*
ADAR	*IL10*	*OSM*
ADGRG1	*IL17A*	*PDGFA*
AHNAK	*IL17RE*	*PDGFB*
AHR	*IL18BP*	*PGF*
ANXA1	*IL1A*	*PTPN22*
AQP3	*IL1R1*	*REL*
AREG	*IL21R*	*RUNX1*
BACH1	*IL23A*	*RUNX2*
BATF	*IL26*	*S100A11*
BATF3	*IL2RG*	*S100A8*
BCL2L1	*IL6R*	*S1PR1*
BTG2	*IL6ST*	*SATB1*
CASP1	*IL9R*	*SGK1*
CASP3	*IRF1*	*SIT1*
CASP8	*IRF3*	*SLAMF1*
CCR6	*IRF7*	*SLC16A1*
CD40LG	*IRF9*	*SLC4A10*
CD68	*ISG15*	*SMAD3*
CEBPD	*ITGA1*	*SOCS1*
CREM	*ITGAL*	*SOCS2*
CSF1	*ITK*	*SOCS3*
DDIT3	*JUND*	*STAT2*
DDX58	*KIR2DL4*	*STING1*
DLL4	*KLRC2*	*TGFA*
FGF1	*KLRD1*	*THEMIS*
FGF22	*LAYN*	*TNFAIP3*
FGF9	*LILRB1*	*TNFRSF18*
FOS	*LILRB2*	*TNFRSF1A*
FOSL2	*MAF*	*TNFRSF1B*
FOXO3	*MAPK1*	*TNFSF9*
FURIN	*MMP25*	*TOX*
GATA3	*MMP28*	*TRBV28*
GBP5	*MR1*	*TRDV2*
GPR171	*MT2A*	*USP18*
GPR183	*MYB*	*VCAM1*
GPR65	*NFAT5*	*VDR*
GZMM	*NFATC1*	*WNT1*
HIF1A	*NFATC2*	*WNT10A*
HOPX	*NFKB1*	*WNT3A*
ID2	*NFKB2*	*WNT6*
ID3	*NFKBIA*	*XCL1*
IFI16	*NFKBIZ*	*XCL2*
IFI27	*NOTCH1*	*ZBP1*
IFIH1	*NR1D1*	*ZBTB32*
IFITM1	*NR3C1*	*ZEB2*
IFNAR1	*NR4A1*	*ZFP36*
IFNAR2	*NR4A2*	
IFNGR2	*NR4A3*	

Supplementary Table S3. List of downregulated genes (all cell types combined) in SD versus non-SD patients. Differential gene expression analysis was performed using a two-sided Wilcoxon rank-sum test (Seurat FindMarkers function). P-values were adjusted for multiple comparisons using Bonferroni correction based on all genes in the Seurat object. N=24 (Non-SD: N=12; SD: N=12).

gene	avg_log2FC	pct.1	pct.2	p_val_adj
ISG15	-1.57966	0.419	0.655	1.3604247083280753e-96
MT2A	-1.11326	0.502	0.713	1.567286568594581e-77
ADAR	-0.75944	0.731	0.798	6.700888579606051e-42
CXCL10	-2.7476	0.027	0.125	9.2502553530978e-36
STAT2	-1.01016	0.448	0.558	4.054749159525832e-32
USP18	-1.3997	0.222	0.363	2.2766887842634936e-31
TNFSF10	-1.55967	0.361	0.459	1.4634121293889796e-30
IRF7	-1.17253	0.296	0.423	7.2428025202253235e-28
IFI16	-0.82645	0.467	0.572	2.3877345424736144e-26
FOS	-0.36184	0.94	0.956	4.512322080542571e-26
CCL2	-2.01643	0.028	0.108	2.0329045335334456e-25
LAMP3	-1.64389	0.056	0.151	1.1279257507540959e-23
CD48	-0.42706	0.861	0.877	8.587165353205064e-23
IFIH1	-1.22886	0.16	0.277	2.120019094844042e-22
LGALS9	-0.72835	0.328	0.447	2.1860033174681364e-18
GIMAP2	-0.78155	0.384	0.491	8.068722309019342e-16
DUSP1	-0.27983	0.947	0.965	3.645380249540277e-15
IER3	-1.37956	0.131	0.215	1.918248955580432e-13
LAP3	-0.66574	0.523	0.561	3.5343273866115514e-13
STAT1	-1.44667	0.157	0.237	6.545196682277028e-12
IFITM3	-0.60652	0.492	0.564	1.0801164855794628e-11
IRF9	-0.77291	0.424	0.485	1.409518891603742e-11
FAM65B	-0.45292	0.498	0.595	1.4142462286946276e-11
ZBP1	-0.83209	0.318	0.397	1.871962521619138e-11
IL7R	-0.55511	0.367	0.458	7.025623472782824e-10
OAS1	-1.13365	0.183	0.256	7.883789892343614e-10
IFITM1	-0.73664	0.229	0.315	9.932875089264501e-10
DDX58	-1.49001	0.04	0.091	2.667602827404134e-9
GBP5	-0.90265	0.21	0.284	1.8062972377737617e-8
FYB	-0.31729	0.57	0.66	2.4474485300143227e-8
ZFP36	-0.25987	0.936	0.918	1.1077185424434843e-7
IL2RG	-0.33721	0.748	0.77	5.213397780188954e-7
GIMAP5	-0.52212	0.212	0.293	7.852835086515958e-7
IL1RN	-1.17056	0.089	0.143	1.3034845260051037e-6
EGR3	-0.81286	0.061	0.11	4.184586522246943e-6
IFITM2	-0.37403	0.583	0.625	5.483156484053746e-6
ID2	-0.3081	0.48	0.547	1.35337097025562e-5
CD4	-0.39184	0.307	0.382	3.798707016645441e-5
TNF	-0.7954	0.209	0.267	4.5750764698257245e-5
CCL4	-0.60747	0.312	0.374	7.234502808786648e-5
CX3CR1	-0.8978	0.225	0.279	1.6653041519554325e-4
OSM	-0.86268	0.053	0.091	3.444712150641305e-4
IL12A	-0.29093	0.043	0.016	3.6926251530861894e-4
SELPLG	-0.3313	0.648	0.652	7.577726520062362e-4
BCL2A1	-0.48191	0.21	0.267	0.001042
SOCS2	-0.90943	0.056	0.091	0.003775
TRAT1	-0.68496	0.171	0.219	0.003936
CXCL11	-1.94217	0.007	0.023	0.005333
LAT	-0.36835	0.407	0.453	0.009415
TRAC	-0.25651	0.578	0.621	0.015589
SELL	-0.32676	0.704	0.679	0.017384
NR4A1	-0.41058	0.239	0.289	0.017573
ITK	-0.30858	0.42	0.461	0.024415
LEF1	-0.40127	0.321	0.368	0.026551
IL1A	-2.33252	0.011	0.028	0.02675
IL1B	-0.76319	0.158	0.198	0.02873
AQP9	-0.5235	0.086	0.123	0.040864

Supplementary Table S4. List of reagents and materials used for this study.

REAGENT or RESOURCE	SOURCE	DILUTION (μl)/ CONCENTRATIO	IDENTIFIER
**Antibodies**			
Mouse Anti-Human CD279 (PD-1) (clone EH12.1)	BD Biosciences	1:50	Cat# 612791; RRID:AB_2870118
Mouse Anti-Human CD279 (PD-1) (clone EH12.2H7)	Biolegend	1:50	Cat# 329927; RRID:AB_11218612
Mouse Anti-Human CD274 (PD-L1) (clone 29E.2A3)	Biolegend	0.5:50	Cat# 329706; RRID:AB_940368
Mouse Anti-Human CD152 (CTLA-4) (clone BNI3)	Biolegend	2.5:50	Cat# 369615; RRID:AB_2632877
Mouse Anti-Human CD223 (LAG-3) (clone 3DS223H)	eBioscience	1:50	Cat# 25-2239-41; RRID:AB_2573429
Mouse Anti-Human CD223 (LAG-3) (clone 11C3C65)	Biolegend	1:50	Cat# 369343; RRID:AB_2910416
Mouse Anti-Human CD366 (TIM-3) (clone F38-2E2)	eBioscience	2.5:50	Cat# 63-3109-42; RRID:AB_2688208
Mouse Anti-Human CD366 (TIM-3) (clone F38-2E2)	Biolegend	1:50	Cat# 345013; RRID:AB_2561719
Mouse Anti-Human TIGIT (clone MBSA43)	eBioscience	1.67:50	Cat# 46-9500-42; RRID:AB_10853679
Mouse Anti-Human TIGIT (clone A15153G)	Biolegend	0.5:50	Cat# 372733; RRID:AB_2876700
Mouse Anti-Human CD69 (clone FN50)	Biolegend	5:50	Cat# 310931; RRID:AB_2561370
Mouse Anti-Human CD69 (clone FN50)	BD Biosciences	0.5:50	Cat# 750214; RRID:AB_2874415
Mouse Anti-Human CD56 (clone HCD56)	Biolegend	0.5:50	Cat# 318336; RRID:AB_2562417
Mouse Anti-Human CD56 (clone NCAM16.2)	BD Biosciences	0.5:50	Cat# 564849; RRID:AB_2738983
Mouse Anti-Human CD56 (clone 5.1H11)	Biolegend	1:50	Cat# 362549; RRID:AB_2566058
Mouse Anti-Human CD16 (clone 3G8)	Biolegend	0.5:50	Cat# 302007; RRID:AB_314207
Mouse Anti-Human CD16 (clone 3G8)	Biolegend	0.2:50	Cat# 302045; RRID:AB_2561367
Mouse Anti-Human CD336 (NKp44) (clone P44-8)	BD Biosciences	2:50	Cat# 744305; RRID:AB_2742135
Mouse Anti-Human CD159C (NKG2C) (clone HP-3D9)	BD Biosciences	1:50	Cat# 749685; RRID:AB_2873941
Mouse Anti-Human CD314 (NKG2D) (clone 1D11)	BD Biosciences	2:50	Cat# 563408; RRID:AB_2738188
Mouse Anti-Human CD85j (LILRB1) (clone GHI/75)	Biolegend	1:50	Cat# 333730
Mouse Anti-Human CD168e1 (KIR3DL1) (clone DX9)	Biolegend	0.5:50	Cat# 312717; RRID:AB_2563361
Mouse Anti-Human CD57 (clone QA17A04)	Biolegend	0.5:50	Cat# 393310; RRID:AB_2750338
Mouse Anti-Human CD335 (NKp46) (clone 9E2)	Biolegend	1:50	Cat# 331908; RRID:AB_1027666

Mouse Anti-Human CD159a (NKG2A) (clone S19004C)	Biolegend	2.5:50	Cat# 375105; RRID:AB_2890806
Mouse Anti-Human CD178 (clone NOK-1)	BD Biosciences	0.5:50	Cat# 744103; RRID:AB_2741997
Mouse Anti-Human CD253 (clone RIK-2)	BD Biosciences	1:50	Cat# 743721; RRID:AB_2741697
Mouse Anti-Human CD226 (DNAM-1) (clone 11A8)	Biolegend	0.5:50	Cat# 338304; RRID:AB_2228763
Mouse Anti-Human IFN-γ (clone B27)	BD Biosciences	3:50	Cat# 560371; RRID:AB_1645594
Mouse Anti-Human TNF-α (clone MAb11)	Biolegend	3:50	Cat# 502946; RRID:AB_2564173
Rat Anti-Human IL-2 (clone MQ1-17H12)	Biolegend	2:50	Cat# 500322; RRID:AB_2264650
Mouse Anti-Human CD107a (LAMP-1) (clone H4A3)	Biolegend	0.75:50	Cat# 328610; RRID:AB_1227504
Mouse Anti-Human CCL4 (MIP-1β) (clone FL34Z3L)	eBioscience	2:50	Cat# 17-7540-42; RRID:AB_2573264
Mouse Anti-Human CD25 (clone M-A251)	BD Biosciences	1.25:50	Cat# 562442; RRID:AB_11154578
Mouse Anti-Human CD25 (clone 2A3)	BD Biosciences	0.3:50	Cat# 564033; RRID:AB_2738555
Rat Anti-Human FOXP3 (clone PCH101)	eBioscience	0.625:50	Cat# 12-4776-42; RRID:AB_1518782
Mouse Anti-Human FOXP3 (clone 259D)	Biolegend	0.625:50	Cat# 320208; RRID:AB_492982
Mouse Anti-Human CD278 (clone DX29)	BD Biosciences	1:50	Cat# 562834; RRID:AB_2737826
Mouse Anti-Human CD195 (clone 2D7/CCR5)	BD Biosciences	5:50	Cat# 565224; RRID:AB_2739120
Mouse Anti-Human CD38 (clone HIT2)	Biolegend	2.5:50	Cat# 303528; RRID:AB_2563811
Mouse Anti-Human HLA-DR (clone L243)	Biolegend	2.5:50	Cat# 307640; RRID:AB_2561913
Zombie Aqua Fixable Viability Kit	Biolegend	1:1000	Cat# 423102
Mouse Anti-Human Ki-67 (clone Ki-67)	Biolegend	3:50	Cat# 350505; RRID:AB_10896915
Rat Anti-Human Ki-67 (clone SolA15))	eBioscience	0.5:50	Cat# 363-5698-82; RRID:AB_2925289
Mouse Anti-Human Perforin (clone B-D48)	Biolegend	1:50	Cat# 353313; RRID:AB_2571970
Mouse Anti-Human Perforin (clone dG9)	Biolegend	2.5:50	Cat# 308129; RRID:AB_2687189
Mouse Anti-Human Granzyme B (clone QA16A02)	Biolegend	1:50	Cat# 372219; RRID:AB_2728386
Rat Anti-Human CLA (clone HECA-452)	Biolegend	10:50	Cat# 321306; RRID:AB_492898
Mouse Anti-Human GPR56 (clone CG4)	Biolegend	2.5:50	Cat# 358205; RRID:AB_2562089
Mouse Anti-Human CD4 (clone RPA-T4)	Biolegend	0.625:50	Cat# 300535; RRID:AB_2561351
Mouse Anti-Human CD8 (clone SK1)	Biolegend	2:50	Cat# 344713; RRID:AB_2044005
Mouse Anti-Human CD3 (clone UCHT1)	BD Biosciences	1:50	Cat# 557943; RRID:AB_396952

Mouse Anti-Human CD3 (clone UCHT1)	BD Biosciences	3:50	Cat# 561416; RRID:AB_10612021
Mouse Anti-Human CD3 (clone UCHT1)	BD Biosciences	2:50	Cat# 560835; RRID:AB_2033956
Mouse Anti-Human CD95 (clone DX2)	BD Biosciences	2:50	Cat# 740306; RRID:AB_2740044
Mouse Anti-Human CD19 (clone HIB19)	Biolegend	0.5:50	Cat# 302241; RRID:AB_2561381
Mouse Anti-Human CD14 (clone M5E2)	Biolegend	0.5:50	Cat# 301841; RRID:AB_2561379
Mouse Anti-Human CD28 (clone CD28.2)	Biolegend	3:50	Cat# 302968; RRID:AB_2800755
Mouse Anti-Human CD27 (clone O323)	Biolegend	1:50	Cat# 302831; RRID:AB_11219185
Mouse Anti-Human CD45RA (clone HI100)	BD Biosciences	0.5:50	Cat# 555489; RRID:AB_395880
Mouse Anti-Human CD197 (CCR7) (clone G043H7)	Biolegend	2.5:50	Cat# 353226; RRID:AB_11126145
Mouse Anti-Human Puromycin (clone 12D10)	Sigma-Aldrich	0.25:50	Cat# MABE343
Rabbit Anti-Human GLUT1 (clone EPR3915)	Abcam	1:50	Cat# ab210438; RRID:AB_2895210
Mouse Anti-Human CPT1A (clone 8F6AE9)	Abcam	1:50	Cat# ab171449; RRID:AB_2714024
Rabbit Anti-Human HK1 [clone EPR1134(B)]	Abcam	1:50	Cat# ab303119; RRID:AB_3206297
Rabbit Anti-Human ATP5A [clone EPR13030(B)]	Abcam	1:50	Cat# ab196198; RRID:AB_3224415
*InVivo*SIM Anti-Human PD-1 (clone Nivolumab)	Bio X Cell	10 µg/mL	Cat# SIM0003
*InVivo*SIM Anti-Human PD-L1 (clone Atezolizumab)	Bio X Cell	10 µg/mL	Cat# SIM0009
*InVivo*MAb Human IgG1 isotype control	Bio X Cell	10 µg/mL	Cat# BE0297
RecombiMAb human IgG4 (S228P) isotype control	Bio X Cell	10 µg/mL	Cat# CP147
Mouse Anti-Human CD45 (clone HI30)	Biolegend	1:100	Cat# 982322; RRID:AB_2936522
**Biological samples**			
Human peripheral blood mononuclear cells from dengue patients	Hospital for Tropical Diseases Ho Chi Minh City		CS/BND/19/34
**Chemicals, peptides, and recombinant proteins**			
RPMI 1640	Gibco		Cat# 11-875-093
AIM V	Gibco		Cat# 12055091
Fetal Bovine Serum (FBS)	Gibco		Cat# 10500064
Human serum	Sigma-Aldrich		Cat# H6914
Puromycin dihydrochloride from Streptomyces alboniger	Sigma-Aldrich		Cat# P7255
BD Cytofix/Cytoperm Fixation/Permeabilization Kit	BD Biosciences		Cat# 554714

eBioscience Foxp3/Transcriptional Factor fixation buffer	Invitrogen		Cat# 00-5523-00
eBioscience Foxp3/Transcriptional Factor permeabilization buffer	Invitrogen		Cat# 00-5523-00
DMSO	Sigma-Aldrich		Cat# D8418
Brefeldin A	BD Biosciences		Cat# 347688
Monensin	Invitrogen		Cat# 00-4505-51
PMA	Sigma-Aldrich		Cat# P1585
Ionomycin	Sigma-Aldrich		Cat# I0634
αCD3/CD28 Dynabeads	Gibco		Cat# 11131D
Human IL-12	Miltenyi Biotec		Cat# 130-096-704
Human IL-18	R&D Systems		Cat# 9124-IL/CF
HyClone Phosphate Buffered Saline	Thermo Fisher Scientific		Cat# SH30256.01
Bovine Serum Albumin Fraction V	Sigma-Aldrich		Cat# 3117332001
2-deoxy-D-glucose	Sigma-Aldrich		Cat# D6134
Oligomycin A	Sigma-Aldrich		Cat# 75351
Puromycin	Sigma-Aldrich		Cat# P7255
OneComp eBeads Compensation Beads	Thermo Fisher Scientific		Cat# 01-1111-42
Ultra eBeads Compensation Beads	Thermo Fisher Scientific		Cat# 01-2222-42
NS3 DENV1 - 2016_Singapore_DENV-1_NPHL	Mimotopes		accession#MF314188
NS3 DENV2 - Thailand/16681/84	Mimotopes		accession#NP056776
NS3 DENV3 - SG(EHI)D3/23167Y15	Mimotopes		accession#KY921906
NS3 DENV4 - SG(EHI)D4/09291Y16	Mimotopes		accession#KY921909
Flex Single-Cell Multiplexing Kit A, Flex Sample Tag 1-6	BD Biosciences		Cat# 633849
Flex Single-Cell Multiplexing Kit B, Flex Sample Tag 7-12	BD Biosciences		Cat# 633850
Flex Single-Cell Multiplexing Kit C, Flex Sample Tag 13-18	BD Biosciences		Cat# 633851
Flex Single-Cell Multiplexing Kit D, Flex Sample Tag 19-24	BD Biosciences		Cat# 633852
BD Rhapsody™ Cartridge Kit	BD Biosciences		Cat# 633733
BD Rhapsody™ Enhanced Cartridge Reagent Kit	BD Biosciences		Cat# 664887
BD Rhapsody™ Immune Response Panel HS	BD Biosciences		Cat# 633750

BD Pharmingen™ Stain Buffer (FBS)	BD Biosciences		Cat# 554656
Qubit™ dsDNA Quantification Assay Kit	Thermo Fisher Scientific		Cat# Q32851
Human TruStain FcX™ (Fc Receptor Blocking Solution)	Biolegend		Cat# 422301
ProcartaPlex™ Human Inflammation Panel, 20plex	Invitrogen		Cat# EPX200-12185-901
**Experimental models: Cell lines**			
Human: K562	ATCC		ATCC CCL-243

Supplementary data 1. List of all expressed proteins as assessed by TMT proteomics. N=11 (Non-SD: N=6; SD: N=5). Proteomics data were processed in Perseus v2.0.7.0. Group comparisons were performed with two-sided Welch's t-test (unequal variances) between non-SD and SD patients. The p-values were adjusted by the permutation-based FDR procedure implemented in Perseus v2.0.7.0 (default setting).
Refer to the excel sheet for the dataset.
