# Supplementary material for: Early NK-cell and T-cell dysfunction marks progression to severe dengue in patients with obesity and healthy weight
Source: Nat Commun. 2025 Jul 1;16:5569. doi: 10.1038/s41467-025-60941-9 (PMC12214611; doi:10.1038/s41467-025-60941-9)
Supplement: Supplementary file 3 — Description of Additional Supplementary Files [file 41467_2025_60941_MOESM3_ESM.pdf]

Description of Additional Supplementary Files

File Name: Supplementary Data 1
Description: List of all expressed proteins as assessed by TMT proteomics. N=11 (Non-SD: N=6; SD: N=5). Proteomics data were processed in Perseus v2.0.7.0. Group comparisons were performed with two-sided Welch's t-test (unequal variances) between non- 5 SD and SD patients. The p-values were adjusted by the permutation-based FDR procedure implemented in Perseus v2.0.7.0 (default setting).
