# Supplementary material for: Early NK-cell and T-cell dysfunction marks progression to severe dengue in patients with obesity and healthy weight
Source: Nat Commun. 2025 Jul 1;16:5569. doi: 10.1038/s41467-025-60941-9 (PMC12214611; doi:10.1038/s41467-025-60941-9)
Supplement: Supplementary file 5 — Reporting Summary [file 41467_2025_60941_MOESM5_ESM.pdf]

nature portfolio

Corresponding author(s): Laura Rivino

Last updated by author(s): May 22, 2025

Reporting Summary

Nature Portfolio wishes to improve the reproducibility of the work that we publish. This form provides structure for consistency and transparency in reporting. For further information on Nature Portfolio policies, see our Editorial Policies and the Editorial Policy Checklist.

Statistics

For all statistical analyses, confirm that the following items are present in the figure legend, table legend, main text, or Methods section.

n/a	Confirmed	
☐	☒	The exact sample size (*n*) for each experimental group/condition, given as a discrete number and unit of measurement
☐	☒	A statement on whether measurements were taken from distinct samples or whether the same sample was measured repeatedly
☐	☒	The statistical test(s) used AND whether they are one- or two-sided<br>*Only common tests should be described solely by name; describe more complex techniques in the Methods section.*
☒	☐	A description of all covariates tested
☐	☒	A description of any assumptions or corrections, such as tests of normality and adjustment for multiple comparisons
☐	☒	A full description of the statistical parameters including central tendency (e.g. means) or other basic estimates (e.g. regression coefficient) AND variation (e.g. standard deviation) or associated estimates of uncertainty (e.g. confidence intervals)
☐	☒	For null hypothesis testing, the test statistic (e.g. *F*, *t*, *r*) with confidence intervals, effect sizes, degrees of freedom and *P* value noted<br>*Give P values as exact values whenever suitable.*
☒	☐	For Bayesian analysis, information on the choice of priors and Markov chain Monte Carlo settings
☒	☐	For hierarchical and complex designs, identification of the appropriate level for tests and full reporting of outcomes
☒	☐	Estimates of effect sizes (e.g. Cohen's *d*, Pearson's *r*), indicating how they were calculated

Our web collection on statistics for biologists contains articles on many of the points above.

Software and code

Policy information about availability of computer code

Data collection	No software was used.
Data analysis	R v4.2.1 and v4.4.0; v4.4.2; R Studio v2024.09.0+375; FlowJo v10.10.0; BD Rhapsody Sequence Analysis Pipeline (v2.2.1); Perseus v2.1.3.

For manuscripts utilizing custom algorithms or software that are central to the research but not yet described in published literature, software must be made available to editors and reviewers. We strongly encourage code deposition in a community repository (e.g. GitHub). See the Nature Portfolio guidelines for submitting code & software for further information.

Data

Policy information about availability of data

All manuscripts must include a data availability statement. This statement should provide the following information, where applicable:

- Accession codes, unique identifiers, or web links for publicly available datasets
- A description of any restrictions on data availability
- For clinical datasets or third party data, please ensure that the statement adheres to our policy

Flow cytometry data is available at the flow cytometry repository: ID: FR-FCM-Z8DN; ID: FR-FCM-Z8E3; ID: FR-FCM-Z8EU; ID: FR-FCM-Z8EV and ID: FR-FCM-Z8EG. -- Single cell RNA-seq data is available at Gene Expression Omnibus, accession number GSE280483. Data can be accessed using the following token: exyhkywizpobrat. The mass spectrometry proteomics data have been deposited to the ProteomeXchange Consortium via the PRIDE partner repository with the dataset identifier PXD061694. http://www.ebi.ac.uk/pride. Data can be accessed using the following details: Project accession: PXD061694; Token: IPZadWhQVBnh.

Source Data are provided with this paper.
All patient data is anonymised.

Research involving human participants, their data, or biological material

Policy information about studies with human participants or human data. See also policy information about sex, gender (identity/presentation), and sexual orientation and race, ethnicity and racism.

Reporting on sex and gender	Patients of both female and male sex were recruited. As stated in the methods section, each patient with overweight/obesity was matched 1:1 to a healthy weight patient by age group (10-16; >16-21; >21-26; >26-30), sex, admission ward (general or ICU), and illness phase – febrile (fever days 1-3) or critical (fever days 4-5).
Reporting on race, ethnicity, or other socially relevant groupings	The study includes samples collected from Vietnamese dengue patients recruited at the Hospital for Tropical Diseases, Ho Chi Mihn City, Vietnam.
Population characteristics	After informed consent/assent, hospitalised patients aged 10-30 were recruited into an observational study designed for dengue patients with overweight/obesity and healthy weight at the Hospital for Tropical Diseases, Ho Chi Minh City, Vietnam. All patients had confirmed dengue and ≤72 hours of fever, except for a proportion of severe patients who were admitted in the intensive care units (ICUs) up to day 5 of fever. Each patient with overweight/obesity was matched 1:1 to a healthy weight patient by age group (10-16; >16-21; >21-26; >26-30), sex, admission ward (general or ICU), and illness phase – febrile (fever days 1-3) or critical (fever days 4-5). The enrolment criteria were selected to minimise confounders due to ageing and comorbidities and to maximise recruitment of severe cases. The exclusion criteria included diabetes, hypertension, cardiovascular disease, signs or symptoms of any other acute infectious disease, undernutrition, and pregnancy. Definition of BMI groups for paediatric patients (10-19 years) was based on the WHO obesity definition using BMI-for-age 56; for adult patients (20-30 years) BMI status was defined as follows: individuals with overweight/obesity had a BMI ≥25 kg/m2, while HW patients had a BMI ≤22 kg/m2 but not less than 17 kg/m2.
Recruitment	Patients presenting to the hospital with dengue infection were recruited into the study after informed consent. Inclusion and exclusion criteria applied as detailed above.
Ethics oversight	The study protocol, consent and assent forms, and patient information sheets were approved by the ethics committees at the Hospital for Tropical Diseases in Ho Chi Minh City (CS/BND/19/34), the Ministry of Health in Vietnam (24/CN-HĐĐĐ) and the Oxford Tropical Research Ethics Committee (REC) (OxREC reference:36-19). All samples were handled in line with the Human Tissue Act, and research was conducted under a Health Research Authority REC approval (reference: 19/LO/1809).

Note that full information on the approval of the study protocol must also be provided in the manuscript.

Field-specific reporting

Please select the one below that is the best fit for your research. If you are not sure, read the appropriate sections before making your selection.

[x] Life sciences [ ] Behavioural & social sciences [ ] Ecological, evolutionary & environmental sciences

For a reference copy of the document with all sections, see nature.com/documents/nr-reporting-summary-flat.pdf

Life sciences study design

All studies must disclose on these points even when the disclosure is negative.

Sample size	Sample size of 150 patients to be recruited into the observational study was calculated as follows. The sample size of 75 patients per group (150 patients in total) was based on clinical judgement and feasibility considerations. Regarding potential effect on endothelial function (a measure of dengue disease severity, the sample size of 75 patients per group will ensure a 80% power to detect a absolute difference of at least 0.023 (log2 VCAM concentration per day) in the slope of change in VCAM concentration overtime (data was based on a previous study - "An Investigation into the pathophysiology of disease progression in dengue in Vietnam" - (OxTREC 1030-13) on biomarkers of endothelial function in dengue.
Data exclusions	In this immunological study we included all samples for which we had sufficient number of PBMCs to perform the described assays.
Replication	Each finding is reproduced using samples from different patients (biological replicates)- information of the number of patients is included in the figure legend.
Randomization	This is an observational study for which patients were assigned to the normal weight or overweight/obese groups as defined in the material and methods section (based on BMI/weight for height). Dengue disease severity was also defined based on WHO guidelines as described in the material and methods section. Hence no randomization was performed for this study which does not include any interventions.
Blinding	In designing experiments we did not perform blinding to ensure representation of patients from all groups. However researchers were blinded during data analyses to avoid bias; analysed data was subsequently stratfied by patient groups (disease severity and/or BMI groups).

Reporting for specific materials, systems and methods

We require information from authors about some types of materials, experimental systems and methods used in many studies. Here, indicate whether each material, system or method listed is relevant to your study. If you are not sure if a list item applies to your research, read the appropriate section before selecting a response.

Materials & experimental systems

n/a	Involved in the study
☐	☒ Antibodies
☐	☒ Eukaryotic cell lines
☐	☐ Palaeontology and archaeology
☐	☐ Animals and other organisms
☐	☒ Clinical data
☐	☐ Dual use research of concern
☐	☐ Plants

Methods

n/a	Involved in the study
☐	☐ ChIP-seq
☐	☒ Flow cytometry
☐	☐ MRI-based neuroimaging

Antibodies

Antibodies used

Mouse Anti-Human CD279 (PD-1) (clone EH12.1) BD Biosciences Cat# 612791; RRID:AB_2870118
Mouse Anti-Human CD279 (PD-1) (clone EH12.2H7) Biolegend Cat# 329927; RRID:AB_11218612
Mouse Anti-Human CD274 (PD-L1) (clone 29E.2A3) Biolegend Cat# 329706; RRID:AB_940368
Mouse Anti-Human CD152 (CTLA-4) (clone BNI3) Biolegend Cat# 369615; RRID:AB_2632877
Mouse Anti-Human CD223 (LAG-3) (clone 3DS223H) eBioscience Cat# 25-2239-41; RRID:AB_2573429
Mouse Anti-Human CD223 (LAG-3) (clone 11C3C65) Biolegend Cat# 369343; RRID:AB_2910416
Mouse Anti-Human CD366 (TIM-3) (clone F38-2E2) eBioscience Cat# 63-3109-42; RRID:AB_2688208
Mouse Anti-Human CD366 (TIM-3) (clone F38-2E2) Biolegend Cat# 345013; RRID:AB_2561719
Mouse Anti-Human TIGIT (clone MBSA43) eBioscience Cat# 46-9500-42; RRID:AB_10853679
Mouse Anti-Human TIGIT (clone A15153G) Biolegend Cat# 372733; RRID:AB_2876700
Mouse Anti-Human CD69 (clone FN50) Biolegend Cat# 310931; RRID:AB_2561370
Mouse Anti-Human CD69 (clone FN50) BD Biosciences Cat# 750214; RRID:AB_2874415
Mouse Anti-Human CD56 (clone HCD56) Biolegend Cat# 318336; RRID:AB_2562417
Mouse Anti-Human D56 (clone NCAM16.2) BD Biosciences Cat# 564849; RRID:AB_2738983
Mouse Anti-Human CD56 (clone 5.1H11) Biolegend Cat# 362549; RRID:AB_2566058
Mouse Anti-Human CD16 (clone 3G8) Biolegend Cat# 302007; RRID:AB_314207
Mouse Anti-Human CD16 (clone 3G8) Biolegend Cat# 302045; RRID:AB_2561367
Mouse Anti-Human CD336 (NKp44) (clone P44-8) BD Biosciences Cat# 744305; RRID:AB_2742135
Mouse Anti-Human CD159C (NKG2C) (clone HP-3D9) BD Biosciences Cat# 749685; RRID:AB_2873941
Mouse Anti-Human CD314 (NKG2D) (clone 1D11) BD Biosciences Cat# 563408; RRID:AB_2738188
Mouse Anti-Human CD85j (LILRB1) (clone GHI/75) Biolegend Cat# 333730
Mouse Anti-Human CD168e1 (KIR3DL1) (clone DX9) Biolegend Cat# 312717; RRID:AB_2563361
Mouse Anti-Human CD57 (clone QA17A04) Biolegend Cat# 393310; RRID:AB_2750338
Mouse Anti-Human CD335 (NKp46) (clone 9E2) Biolegend Cat# 331908; RRID:AB_1027666
Mouse Anti-Human CD159a (NKG2A) (clone S19004C) Biolegend Cat# 375105; RRID:AB_2890806
Mouse Anti-Human CD178 (clone NOK-1) BD Biosciences Cat# 744103; RRID:AB_2741997
Mouse Anti-Human CD253 (clone RIK-2) BD Biosciences Cat# 743721; RRID:AB_2741697
Mouse Anti-Human CD226 (DNAM-1) (clone 11A8) Biolegend Cat# 338304; RRID:AB_2228763
Mouse Anti-Human IFN-γ (clone B27) BD Biosciences Cat# 560371; RRID:AB_1645594
Mouse Anti-Human TNF-α (clone MAb11) Biolegend Cat# 502946; RRID:AB_2564173
Rat Anti-Human IL-2 (clone MQ1-17H12) Biolegend Cat# 500322; RRID:AB_2264650
Mouse Anti-Human CD107a (LAMP-1) (clone H4A3) Biolegend Cat# 328610; RRID:AB_1227504
Mouse Anti-Human CCL4 (MIP-1β) (clone FL34Z3L) eBioscience Cat# 17-7540-42; RRID:AB_2573264
Mouse Anti-Human CD25 (clone M-A251) BD Biosciences Cat# 562442; RRID:AB_11154578
Mouse Anti-Human CD25 (clone 2A3) BD Biosciences Cat# 564033; RRID:AB_2738555
Rat Anti-Human FOXP3 (clone PCH101) eBioscience Cat# 12-4776-42; RRID:AB_1518782
Mouse Anti-Human FOXP3 (clone 259D) Biolegend Cat# 320208; RRID:AB_492982
Mouse Anti-Human CD278 (clone DX29) BD Biosciences Cat# 562834; RRID:AB_2737826
Mouse Anti-Human CD195 (clone 2D7/CCR5) BD Biosciences Cat# 565224; RRID:AB_2739120
Mouse Anti-Human CD38 (clone HIT2) Biolegend Cat# 303528; RRID:AB_2563811
Mouse Anti-Human HLA-DR (clone L243) Biolegend Cat# 307640; RRID:AB_2561913
Zombie Aqua Fixable Viability Kit Biolegend Cat# 423102
Mouse Anti-Human Ki-67 (clone Ki-67) Biolegend Cat# 350505; RRID:AB_10896915
Rat Anti-Human Ki-67 (clone SolA15)) eBioscience Cat# 363-5698-82; RRID:AB_2925289
Mouse Anti-Human Perforin (clone B-D48) Biolegend Cat# 353313; RRID:AB_2571970
Mouse Anti-Human Perforin (clone dG9) Biolegend Cat# 308129; RRID:AB_2687189

Mouse Anti-Human Granzyme B (clone QA16A02) Biolegend Cat# 372219; RRID:AB_2728386
Rat Anti-Human CLA (clone HECA-452) Biolegend Cat# 321306; RRID:AB_492898
Mouse Anti-Human GPR56 (clone CG4) Biolegend Cat# 358205; RRID:AB_2562089
Mouse Anti-Human CD4 (clone RPA-T4) Biolegend Cat# 300535; RRID:AB_2561351
Mouse Anti-Human CD8 (clone SK1) Biolegend Cat# 344713; RRID:AB_2044005
Mouse Anti-Human CD3 (clone UCHT1) BD Biosciences Cat# 557943; RRID:AB_396952
Mouse Anti-Human CD3 (clone UCHT1) BD Biosciences Cat# 561416; RRID:AB_10612021
Mouse Anti-Human CD3 (clone UCHT1) BD Biosciences Cat# 560835; RRID:AB_2033956
Mouse Anti-Human CD95 (clone DX2) BD Biosciences Cat# 740306; RRID:AB_2740044
Mouse Anti-Human CD19 (clone HIB19) Biolegend Cat# 302241; RRID:AB_2561381
Mouse Anti-Human CD14 (clone M5E2) Biolegend Cat# 301841; RRID:AB_2561379
Mouse Anti-Human CD28 (clone CD28.2) Biolegend Cat# 302968; RRID:AB_2800755
Mouse Anti-Human CD27 (clone O323) Biolegend Cat# 302831; RRID:AB_11219185
Mouse Anti-Human CD45RA (clone HI100) BD Biosciences Cat# 555489; RRID:AB_395880
Mouse Anti-Human CD197 (CCR7) (clone G043H7) Biolegend Cat# 353226; RRID:AB_11126145
Mouse Anti-Human Puromycin (clone 12D10) Sigma-Aldrich Cat# MABE343
Rabbit Anti-Human GLUT1 (clone EPR3915) Abcam Cat# ab210438; RRID:AB_2895210
Mouse Anti-Human CPT1A (clone 8F6AE9) Abcam Cat# ab171449; RRID:AB_2714024
Rabbit Anti-Human HK1 [clone EPR1134(B)] Abcam Cat# ab303119; RRID:AB_3206297
Rabbit Anti-Human ATP5A [clone EPR13030(B)] Abcam Cat# ab196198; RRID:AB_3224415
InVivoSIM Anti-Human PD-1 (clone Nivolumab) Bio X Cell Cat# SIM0003
InVivoSIM Anti-Human PD-L1 (clone Atezolizumab) Bio X Cell Cat# SIM0009
InVivoMAb Human IgG1 isotype control Bio X Cell Cat# BE0297
RecombiMAb human IgG4 (S228P) isotype control Bio X Cell Cat# CP147
Mouse Anti-Human CD45 (clone HI30) Biolegend Cat# 982322; RRID:AB_2936522

Validation: Flow cytometry - titration using human PBMCs

Eukaryotic cell lines

Policy information about cell lines and Sex and Gender in Research

Cell line source(s)	Human K562 ATCC (ATCC CCL-243)
Authentication	Cell line was bought from ATCC
Mycoplasma contamination	Cell line was mycoplasma negative
Commonly misidentified lines (See ICLAC register)	NA

Palaeontology and Archaeology

Specimen provenance	*Provide provenance information for specimens and describe permits that were obtained for the work (including the name of the issuing authority, the date of issue, and any identifying information). Permits should encompass collection and, where applicable, export.*
Specimen deposition	*Indicate where the specimens have been deposited to permit free access by other researchers.*
Dating methods	*If new dates are provided, describe how they were obtained (e.g. collection, storage, sample pretreatment and measurement), where they were obtained (i.e. lab name), the calibration program and the protocol for quality assurance OR state that no new dates are provided.*

☐ Tick this box to confirm that the raw and calibrated dates are available in the paper or in Supplementary Information.

Ethics oversight	*Identify the organization(s) that approved or provided guidance on the study protocol, OR state that no ethical approval or guidance was required and explain why not.*

Note that full information on the approval of the study protocol must also be provided in the manuscript.

Animals and other research organisms

Policy information about studies involving animals; ARRIVE guidelines recommended for reporting animal research, and Sex and Gender in Research

Laboratory animals	NA
Wild animals	*Provide details on animals observed in or captured in the field; report species and age where possible. Describe how animals were caught and transported and what happened to captive animals after the study (if killed, explain why and describe method; if released, say where and when) OR state that the study did not involve wild animals.*
Reporting on sex	*Indicate if findings apply to only one sex; describe whether sex was considered in study design, methods used for assigning sex. Provide data disaggregated for sex where this information has been collected in the source data as appropriate; provide overall numbers in this Reporting Summary. Please state if this information has not been collected. Report sex-based analyses where performed, justify reasons for lack of sex-based analysis.*
Field-collected samples	*For laboratory work with field-collected samples, describe all relevant parameters such as housing, maintenance, temperature, photoperiod and end-of-experiment protocol OR state that the study did not involve samples collected from the field.*
Ethics oversight	*Identify the organization(s) that approved or provided guidance on the study protocol, OR state that no ethical approval or guidance was required and explain why not.*

Note that full information on the approval of the study protocol must also be provided in the manuscript.

Clinical data

Policy information about clinical studies
All manuscripts should comply with the ICMJEguidelines for publication of clinical research and a completedCONSORT checklist must be included with all submissions.

Clinical trial registration	NA
Study protocol	*Note where the full trial protocol can be accessed OR if not available, explain why.*
Data collection	*Describe the settings and locales of data collection, noting the time periods of recruitment and data collection.*
Outcomes	*Describe how you pre-defined primary and secondary outcome measures and how you assessed these measures.*

Dual use research of concern

Policy information about dual use research of concern

Hazards

Could the accidental, deliberate or reckless misuse of agents or technologies generated in the work, or the application of information presented in the manuscript, pose a threat to:

No	Yes	
☒	☐	Public health
☒	☐	National security
☒	☐	Crops and/or livestock
☒	☐	Ecosystems
☒	☐	Any other significant area

Experiments of concern

Does the work involve any of these experiments of concern:

No	Yes	
☒	☐	Demonstrate how to render a vaccine ineffective
☒	☐	Confer resistance to therapeutically useful antibiotics or antiviral agents
☒	☐	Enhance the virulence of a pathogen or render a nonpathogen virulent
☒	☐	Increase transmissibility of a pathogen
☒	☐	Alter the host range of a pathogen
☒	☐	Enable evasion of diagnostic/detection modalities
☒	☐	Enable the weaponization of a biological agent or toxin
☒	☐	Any other potentially harmful combination of experiments and agents

Plants

Seed stocks	NA
Novel plant genotypes	NA
Authentication	*Describe any authentication procedures for each seed stock used or novel genotype generated. Describe any experiments used to assess the effect of a mutation and, where applicable, how potential secondary effects (e.g. second site T-DNA insertions, mosiacism, off-target gene editing) were examined.*

ChIP-seq

Data deposition

☐ Confirm that both raw and final processed data have been deposited in a public database such as GEO.

☐ Confirm that you have deposited or provided access to graph files (e.g. BED files) for the called peaks.

Data access links<br>*May remain private before publication.*	*For "Initial submission" or "Revised version" documents, provide reviewer access links. For your "Final submission" document, provide a link to the deposited data.*
Files in database submission	*Provide a list of all files available in the database submission.*
Genome browser session<br>(e.g. UCSC)	*Provide a link to an anonymized genome browser session for "Initial submission" and "Revised version" documents only, to enable peer review. Write "no longer applicable" for "Final submission" documents.*

Methodology

Replicates	*Describe the experimental replicates, specifying number, type and replicate agreement.*
Sequencing depth	*Describe the sequencing depth for each experiment, providing the total number of reads, uniquely mapped reads, length of reads and whether they were paired- or single-end.*
Antibodies	*Describe the antibodies used for the ChIP-seq experiments; as applicable, provide supplier name, catalog number, clone name, and lot number.*
Peak calling parameters	*Specify the command line program and parameters used for read mapping and peak calling, including the ChIP, control and index files used.*
Data quality	*Describe the methods used to ensure data quality in full detail, including how many peaks are at FDR 5% and above 5-fold enrichment.*
Software	*Describe the software used to collect and analyze the ChIP-seq data. For custom code that has been deposited into a community repository, provide accession details.*

Flow Cytometry

Plots

Confirm that:

- [ ] The axis labels state the marker and fluorochrome used (e.g. CD4-FITC).
- [x] The axis scales are clearly visible. Include numbers along axes only for bottom left plot of group (a 'group' is an analysis of identical markers).
- [x] All plots are contour plots with outliers or pseudocolor plots.
- [x] A numerical value for number of cells or percentage (with statistics) is provided.

Methodology

Sample preparation	Frozen human PBMCs, PBMCs were rapidly thawed in RPMI 10% FBS, washed 2x with PBS 1% BSA before seeding
Instrument	BD Fortessa X20
Software	FlowJo v10.10.0; R v4.2.1
Cell population abundance	NA
Gating strategy	1) Quality control using PeacoQC 2) FSC-A/SSC-A - Lymphocytes 3) FSC-A/FSC-H - Single cells 4) Livedead/FSC-A - Live cells 5) CD3/FSC-A - CD3+/CD3- 6) CD4/CD8<br>We used Fluorescent minus one controls or unstained samples to gate on positive/negative populations and isotype control to check for unspecific binding where necessary.

- [x] Tick this box to confirm that a figure exemplifying the gating strategy is provided in the Supplementary Information.

Magnetic resonance imaging

Experimental design

Design type	*Indicate task or resting state; event-related or block design.*
Design specifications	*Specify the number of blocks, trials or experimental units per session and/or subject, and specify the length of each trial or block (if trials are blocked) and interval between trials.*
Behavioral performance measures	*State number and/or type of variables recorded (e.g. correct button press, response time) and what statistics were used to establish that the subjects were performing the task as expected (e.g. mean, range, and/or standard deviation across subjects).*

Acquisition

Imaging type(s)	*Specify: functional, structural, diffusion, perfusion.*
Field strength	*Specify in Tesla*
Sequence & imaging parameters	*Specify the pulse sequence type (gradient echo, spin echo, etc.), imaging type (EPI, spiral, etc.), field of view, matrix size, slice thickness, orientation and TE/TR/flip angle.*
Area of acquisition	*State whether a whole brain scan was used OR define the area of acquisition, describing how the region was determined.*

Diffusion MRI ☐ Used ☐ Not used

Preprocessing

Preprocessing software	*Provide detail on software version and revision number and on specific parameters (model/functions, brain extraction, segmentation, smoothing kernel size, etc.).*
Normalization	*If data were normalized/standardized, describe the approach(es): specify linear or non-linear and define image types used for transformation OR indicate that data were not normalized and explain rationale for lack of normalization.*
Normalization template	*Describe the template used for normalization/transformation, specifying subject space or group standardized space (e.g. original Talairach, MNI305, ICBM152) OR indicate that the data were not normalized.*
Noise and artifact removal	*Describe your procedure(s) for artifact and structured noise removal, specifying motion parameters, tissue signals and physiological signals (heart rate, respiration).*

Volume censoring | *Define your software and/or method and criteria for volume censoring, and state the extent of such censoring.*

Statistical modeling & inference

Model type and settings | *Specify type (mass univariate, multivariate, RSA, predictive, etc.) and describe essential details of the model at the first and second levels (e.g. fixed, random or mixed effects; drift or auto-correlation).*

Effect(s) tested | *Define precise effect in terms of the task or stimulus conditions instead of psychological concepts and indicate whether ANOVA or factorial designs were used.*

Specify type of analysis: ☐ Whole brain ☐ ROI-based ☐ Both

Statistic type for inference | *Specify voxel-wise or cluster-wise and report all relevant parameters for cluster-wise methods.*

(See Eklund et al. 2016)

Correction | *Describe the type of correction and how it is obtained for multiple comparisons (e.g. FWE, FDR, permutation or Monte Carlo).*

Models & analysis

n/a	Involved in the study
☐	☐ Functional and/or effective connectivity
☐	☐ Graph analysis
☐	☐ Multivariate modeling or predictive analysis

Functional and/or effective connectivity | *Report the measures of dependence used and the model details (e.g. Pearson correlation, partial correlation, mutual information).*

Graph analysis | *Report the dependent variable and connectivity measure, specifying weighted graph or binarized graph, subject- or group-level, and the global and/or node summaries used (e.g. clustering coefficient, efficiency, etc.).*

Multivariate modeling and predictive analysis | *Specify independent variables, features extraction and dimension reduction, model, training and evaluation metrics.*
